# An Isolated Phlebolith on the Lip: An Unusual Case and Review of the Literature

**DOI:** 10.1155/2015/507840

**Published:** 2015-07-22

**Authors:** Gabriela de Morais Gouvêa Lima, Renata Mendonça Moraes, Ana Sueli Rodrigues Cavalcante, Yasmin Rodarte Carvalho, Ana Lia Anbinder

**Affiliations:** Department of Bioscience and Oral Diagnosis, Institute of Science and Technology, Campus São José dos Campos, Univ. Estadual Paulista (UNESP), Engenheiro Francisco José Longo Avenida 777, 12245-000 São José dos Campos, SP, Brazil

## Abstract

*Background*. Calcified thrombi are a common finding, especially in the pelvic veins. There are generally multiple thrombi, and they are generally associated with vascular malformations. *Design*. Herein we report a rare case of a single labial phlebolith, not associated with any other vascular lesion. We aim to alert clinicians to the possibility of the occurrence of vascular thrombi in the mouth and to describe the clinical and histological characteristics of such lesions in order to simplify the diagnosis and treatment. Furthermore, we have reviewed the English-language literature published since 1970 reporting oral (including masticatory muscles) phleboliths. *Results*. Twenty-nine cases of phleboliths have been reported in the literature since 1970. Only three of the reported phleboliths were solitary and not associated with other vascular lesions, as in the case presented here. *Conclusion*. Although phleboliths not associated with other vascular lesions are not common, clinicians should be aware of the existence of this pathology and include it as differential diagnosis of oral lesions.

## 1. Introduction

Phleboliths are calcified thrombi usually associated with other vascular lesions, as vascular malformations (VM), and are caused by blood stasis [1] or even trauma [2].

In the literature, they are commonly found on routine radiographs of cases diagnosed as VM or hemangiomas, although they are better detected by computerized tomography [1, 3, 4]. Radiographically, they present as oval structures with concentric radiolucent or radio-opaque laminations. They can be found in periapical regions near dental structures in a plain radiograph [5, 6]. Nonionizing techniques, such as magnetic resonance imaging and ultrasound, can provide good images demonstrating the location and extent of the lesions [6, 7].

The pelvic area is the most common area affected by phleboliths, which can occur within prostatic, uterine, or intestine veins. Phleboliths are not unusual in the head and neck, but there are few cases in the literature of phleboliths not associated with other vascular anomalies [8–10]. When a phlebolith is located in salivary gland regions, it can be clinically misdiagnosed as a sialolith or salivary gland disease, especially when there is intermittent swelling, despite not being associated with food intake. In these cases, sialography can be used as diagnostic tool [11–13].

Besides imaging exams, biopsy followed by microscopic examination can help in the final diagnosis. Histologically, a phlebolith is seen as concentric calcifications with an onion-like appearance inside a vessel, caused by repetitive mineral deposition on a thrombus and secondary lamellar fibrosis [2, 7, 13]. Commonly, hematoxylin and eosin (HE) and immunohistochemistry are used [10, 14, 15], but use of electron microscopy and X-ray microdiffraction has also been reported [14].

We report herein an unusual case of a phlebolith presenting as a single lesion, not associated with a VM and clinically misdiagnosed as a neurofibroma. In describing this case, we aim to help clinicians to deal with this uncommon but simple lesion. We have also reviewed the literature of oral/masticatory muscle phleboliths.

## 2. Report of Case

A 56-year-old woman was referred to our institution presenting with a white-yellow painless papule in the right labial commissure. It was approximately 3 mm in diameter and firm to palpation and had been growing slowly for approximately one year, following trauma. The patient reported swelling after trauma with subsequent diminution and then a recent increase in volume. Her previous medical history was unremarkable.

No radiograph was performed, and the clinical diagnostic hypothesis was neurofibroma and pleomorphic adenoma. An excisional biopsy was performed followed by histopathological examination.

**Figure 1:**
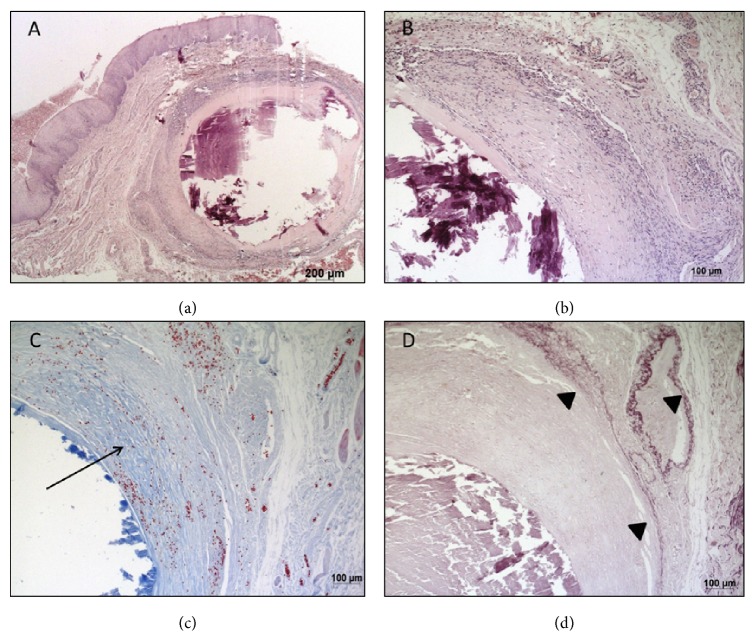
Microscopic aspect of the lesion showing the vascular structures ((a) and (b) hematoxylin and eosin stain), collagen (Masson's Trichrome (c)), and elastic fibers (Weigert stain (d)).

Microscopic examination revealed a submucous dilated vessel filled by a calcified thrombus with a concentric pattern of mineralization and surrounded by granulation tissue and small capillaries under a hyaline layer. The covering mucosa was intact and presented no alterations (Figure 1).

**Figure 2:**
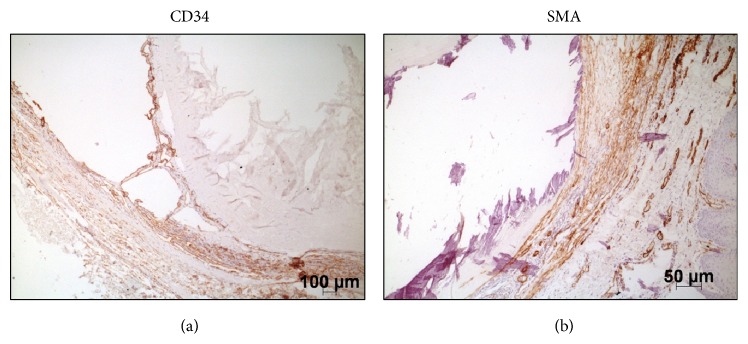
Immunohistochemistry showing vascular endothelium stained with anti-CD34 (a) and smooth muscle wall stained with anti-SMA (b).

The diagnosis of phlebolith was confirmed by histochemical Weigert's and Masson's Trichrome staining and by immunohistochemistry with anti-CD34 and anti-smooth muscle actin (SMA) (Table 1; Figure 2), as evidenced by the vascular endothelium (CD34-positive), smooth muscle wall (SMA-positive), tunica intima elastic fibers (Weigert's elastic stain), and surrounding collagen fibers (Masson's Trichrome) of the vessel filled with the calcified thrombus.

**Figure 3:**
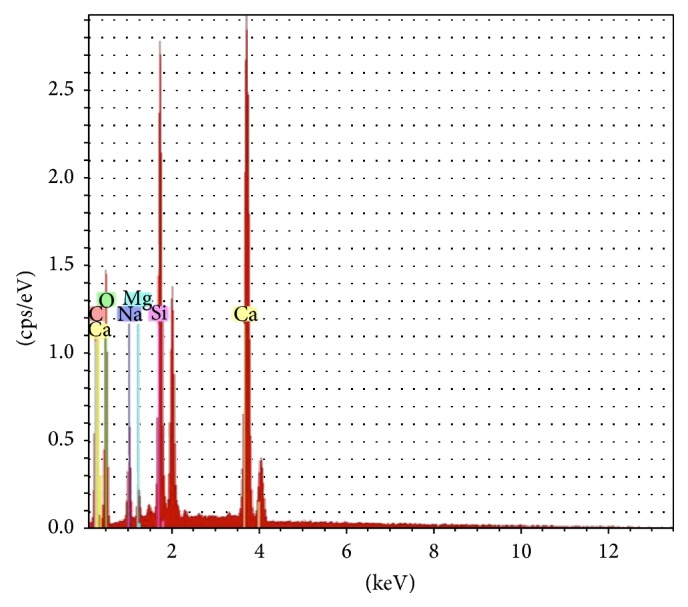
Energy dispersive X-ray spectrometry (EDS), results in graphic, with peaks of calcium, carbon, silicon, and magnesium.

Complementary electron microscopy with energy dispersive X-ray spectrometry (EDS) revealed the presence of calcium (Ca), carbon (C), silicon (Si), and magnesium (Mg) (Figure 3), compatible with a phlebolith. The patient was followed without any complication after surgery.

## 3. Review of the Literature

Medline and Web of Science databases were searched using the descriptors “oral phlebolith” and “phleboliths,” “phlebolith,” “oral thrombus [case report],” and “lip and thrombus.” Table 2 shows all cases of phleboliths in the oral region (including masticatory muscles) reported in the English literature from 1970 to 2013.

We found 29 cases of phleboliths. In five cases, there were multiple phleboliths not associated with other vascular lesions (16.13%), and, in four cases (12.90%), there was a solitary phlebolith, but only one was associated with a lesion diagnosed as hemangioma. Only three reported phleboliths were solitary and not associated with other vascular lesions, as in the case presented here.

Based on the reviewed literature, phleboliths of the oral region can be found in infants to elderly individuals, but mainly between the first and third decades of life (55.2%), with no sex predilection (48.28% female: 51.72% male), and are sometimes associated with masticatory muscles (27.6%).

## 4. Discussion

Phleboliths are calcified thrombi that commonly appear as multiple calcifications related to lesions diagnosed as VM or hemangiomas [1–4, 6, 7, 11–21]. They are rarely reported as solitary calcifications and/or not associated with a vascular lesion [8–10], as in the case reported here. We have reported herein a case of a single phlebolith on the lip, probably caused by trauma. The clinical appearance of a white-yellow color papule, without a calcified aspect or consistency, led us to suspect a neurofibroma or a small salivary gland lesion; therefore, we did not radiograph the lesion, which could have helped us with an accurate diagnosis.

The diagnosis of a phlebolith is commonly made by physical and imaging examinations and confirmed by microscopy (HE stain). In some cases, immunohistochemistry or even electron microscopy [10, 14, 15] is used. In our case, we reached the final diagnosis only after biopsy, and it was confirmed by histochemistry (Weigert's elastic stain and Masson's Trichrome), immunohistochemistry (CD34 and SMA), and EDS.

The EDS showed peaks of C, Ca (interpreted as calcium carbonate), Mg, and Si. A study of arterial biomineralization in autopsies found both apatitic and nonapatitic calcifications in different zones of the arterial wall [22]. The literature shows that the calcified crystals are mainly composed of phosphate and calcium carbonate, components of apatite. Minor crystals of Mg can be present in the hydroxyapatite [11, 17], and even Si may be present. Studies have shown that Si is a component of biomineralization [22], which was recently described as being involved in the early stages of this process [23]. The mineral composition, though phosphorus was missing, is intriguing; however, the components that should be present in a phlebolith are not well established, and the area we analyzed using EDS may not have been a representative area of apatitic calcification.

Cases of phleboliths not associated with other vascular lesions are rarely reported in the literature, especially as solitary nodules [8–10]. Trauma may have caused the slow flow, thrombus development, and consequent mineral deposition in this case.

## 5. Conclusion

Despite the rarity of this condition, clinicians should be aware of the existence of single phleboliths not associated with other vascular lesions when facing a submucous lesion on the lip or other regions susceptible to trauma and should include them in the differential diagnosis.

## Figures and Tables

**Table 1:** Antibodies and protocol used for immunohistochemistry.

Antibody	Antigen retrieval	Concentration
SMA (Dako)	No retrieval	1 : 100
CD34 (Dako)	Dako Target Retrieval Solution, pH 9	1 : 100

**Table 2:** Cases of phleboliths in the oral region (including masticatory muscles) reported in the English literature from 1970 to 2013.

Author	Year	Location	Multiple	Other vascular lesions	Sex	Age
O'Riordan [11]	1974	Lower lip/neck	Yes	Yes	Female	23
O'Riordan [11]	1974	Buccal soft tissue	Yes	Yes	Male	43
O'Riordan [11]	1974	Mandible	Yes	Yes	Female	27
O'Riordan [11]	1974	Buccal soft tissue	Yes	Yes	Female	47
Keathley et al. [12]	1983	Submandibular	Yes	Yes	Male	23
Ikegami and Nishijima [16]	1984	Buccal soft tissue	Yes	Yes	Male	23
Gunge et al. [14]	1987	Buccinator muscle	Yes	Yes	Female	41
Sano et al. [17]	1988	Buccal soft tissue	Yes	Yes	Male	17
Sano et al. [17]	1988	Buccal soft tissue	Yes	Yes	Female	37
Hupp [18]	1989	Maxilla	Yes	Yes	Male	20
Zachariades et al. [8]	1991	Masseteric muscle	Yes	No	Male	8
Zachariades et al. [8]	1991	Masseteric muscle	Yes	No	Female	9
Zachariades et al. [8]	1991	Maxillary antrum	No	No	Male	42
Kurita et al. [9]	1994	Tongue	No	No	Female	69
Scolozzi et al. [13]	2003	Retromolar region	Yes	Yes	Female	92
Çankaya et al. [19]	2003	Submental	Yes	Yes	Male	18
Chuang et al. [3]	2005	Submandibular	Yes	Yes	Male	65
Altuğ et al. [1]	2007	Masseteric muscle	No	Yes	Male	21
Altuğ et al. [1]	2007	Buccal soft tissue	Yes	Yes	Male	22
Altuğ et al. [1]	2007	Submental/submandibular/neck	Yes	Yes	Male	21
Kanaya et al. [7]	2008	Masseteric muscle	Yes	Yes	Female	14
Su et al. [20]	2009	Submandibular	Yes	Yes	Female	26
Mandel and Perrino [2]	2010	Buccinator/masseter	Yes	Yes	Female	56
Mandel and Perrino [2]	2010	Tongue	Yes	Yes	Female	64
Gharaibeh et al. [15]	2010	Floor of the mouth	Yes	Yes	Male	11
Orhan et al. [4]	2012	Lower and upper lip/buccal soft tissue	Yes	Yes	Male	20
Kato et al. [10]	2012	Buccal soft tissue	No	No	Female	17
Zengin et al. [6]	2013	Masseteric muscle	Yes	Yes	Female	21
